# Development and validation of a nomogram for predicting the prognosis in cancer patients with sepsis

**DOI:** 10.1002/cam4.4618

**Published:** 2022-02-18

**Authors:** Yong Yang, Jun Dong, Yang Li, Renxiong Chen, Xiuyun Tian, Hongzhi Wang, Chunyi Hao

**Affiliations:** ^1^ Key Laboratory of Carcinogenesis and Translational Research (Ministry of Education/Beijing), Department of Critical Care Medicine Peking University Cancer Hospital and Institute Beijing People’s Republic of China; ^2^ Key Laboratory of Carcinogenesis and Translational Research (Ministry of Education/Beijing), Department of Hepato‐Pancreato‐Biliary Surgery Peking University Cancer Hospital and Institute Beijing People’s Republic of China

**Keywords:** cancer patients, ICU, mortality, nomogram, sepsis

## Abstract

**Background:**

To develop a multiparameter‐based, easy‐to‐use nomogram and to predict the prognosis of cancer patients with sepsis in the intensive care unit (ICU).

**Methods:**

Clinical data on cancer patients with sepsis who met the definition of sepsis 3.0 admitted to the ICU from January 2016 to October 2021 were collected. All patients were randomly entered into the development cohort or validation cohort according to the ratio of 7:3. Patients in the development cohort were divided into the survivors and the nonsurvivors according to the outcome of 28 days in ICU. The independent risk factors of mortality due to sepsis were screened out from the two groups (the survivors and the nonsurvivors) in the development cohort through multivariate logistic regression analysis. A nomogram was established with these independent risk factors, and the calibration plot was subsequently evaluated. Finally, the predictive power of the nomogram was verified in the validation cohort.

**Results:**

A total of 317 cancer patients with sepsis who met the requirements were enrolled in this study, of which 229 entered into the development cohort and 88 entered into the validation cohort. The 28‐day mortality rates of the two cohorts were 17.5% and 20.5%, respectively. The neutrophil‐to‐lymphocyte ratio (NLR) on day 3 (d3), brain natriuretic peptide (BNP) d3, fluid accumulation at 72 hours (h), and Sequential Organ Failure Assessment (SOFA) score were independent risk factors for the 28‐day mortality between the survivors and the nonsurvivors in the development cohort. A nomogram was established on the above variables. The calibration plots fit well with the nomogram and had good statistical consistency in predicting the 28‐day mortality of sepsis (the *C* value was 0.938 and 0.968 in the two cohorts, respectively). With a nomogram score of 83.8 points, the diagnostic accuracy was 90.8% vs 92.0%, the sensitivity was 72.5% vs 77.7%, the specificity was 94.7% vs 95.7%, the positive predictive value was 72.3% vs 82.4%, and the negative predictive value was 94.2% vs 94.4% for predicting the 28‐day mortality in the development cohort and the validation cohort, respectively.

**Conclusion:**

This easy‐to‐use nomogram based on NLR d3, BNP d3, and fluid accumulation at 72 h and SOFA score provides an accurate 28‐day prognosis prediction for cancer patients with sepsis admitted to the ICU.

## 1 INTRODUCTION

The latest definition of sepsis is an acute organ dysfunction caused by the unbalanced response of the host to infection, which may be life‐threatening in severe cases.^1^, ^2^ In the past 20 years, despite significant progress in treatment technology, the mortality rate of sepsis has remained high. According to statistics, there are 750,000 new sepsis patients in the United States each year, with a mortality rate of 28%.^3^ The incidence of sepsis in ICU patients in China is 20%, and the 90‐day all‐cause mortality rate exceeds 30%.^4^ Cancer patients are more apt to develop sepsis than noncancer patients and have a more serious clinical course and a worse prognosis.^5^

The latest definition of sepsis reflects its inherent heterogeneity. This heterogeneity is reflected in many aspects, including the different causes of infection, the uniqueness of body complications and genetics, and the timeliness of diagnosis and treatment vary from person to person.^6^, ^7^ The above factors will not only affect the evolution of sepsis in individual patients but also have different responses to treatment interventions. Because of the heterogeneity of sepsis, it is difficult to judge the prognosis of sepsis timely and accurately. Searching for effective biomarkers for assessing the prognosis of sepsis has become a hot research institute. At present, more than 170 biomarkers have been studied for patients with sepsis, but only a few of them have been tested for effectiveness and clinical applicability, including procalcitonin (PCT), cluster of differentiation‐14(CD14), CD64, soluble‐urokinase‐type‐Plasminogen‐Activator‐Receptor (suPAR), soluble triggering receptor expressed on myeloid cells 1 (sTREM‐1), and so on. However, the sensitivity or specificity of the above multiple markers in judging the prognosis of sepsis varies greatly in different studies. This may be due to the fact that sepsis is a heterogeneous biological syndrome that is not fully understood.^8^, ^9^ Therefore, few biomarkers combine excellent sensitivity and specificity in the prognostic assessment of sepsis. It is more inclined to combine multiple markers to assess the prognosis of sepsis.^10^, ^11^

We conducted this study to clarify the correlation between multiple clinical variables and the 28‐day mortality of sepsis to find simple and effective variables to construct a prediction model for assessing the severity and judging the prognosis of sepsis reasonably. Meanwhile, a nomogram based on logistic regression was established to assess the predictive power of independent risk factors screened out from the multiple clinical variables for the prognosis of sepsis in cancer patients accurately.

## 2 METHODS

### 2.1 Participants

**FIGURE 1:**
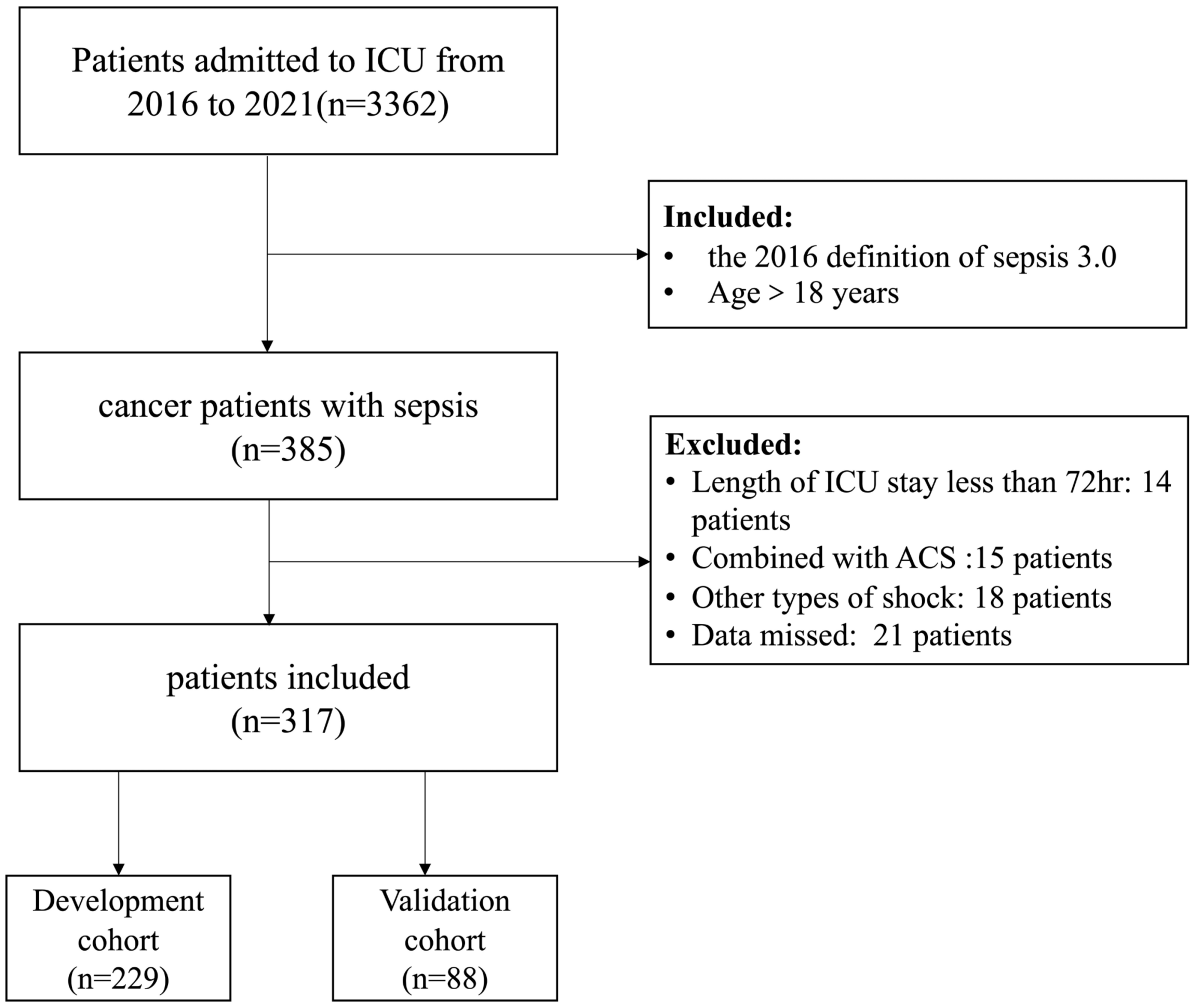
Flow diagrams of studying the selection process

This study was approved by the Ethics Committee of Peking University Cancer Hospital. All patients were issued written informed consent so that their data could be used for scientific purposes. This was a retrospective study of cancer patients with sepsis in the ICU of Peking University Cancer Hospital from January 2016 to October 2021. The inclusion criteria consisted of the following: 1. all patients met the diagnosis based on the sepsis definition 3.0^2^ and 2. patients ≥18 years. The exclusion criteria consisted of the following: (1) life expected <72 h in ICU, (2) acute coronary syndrome (ACS), (3) existence of other types of shock except for septic shock, and (4) incomplete clinical data. All patients were randomly entered into the development cohort or validation cohort according to the ratio of 7:3 (Figure 1). The nomogram based on the development cohort was established and its effectiveness was tested in the validation cohort.

### 2.2 Data collection

**FIGURE 2:**
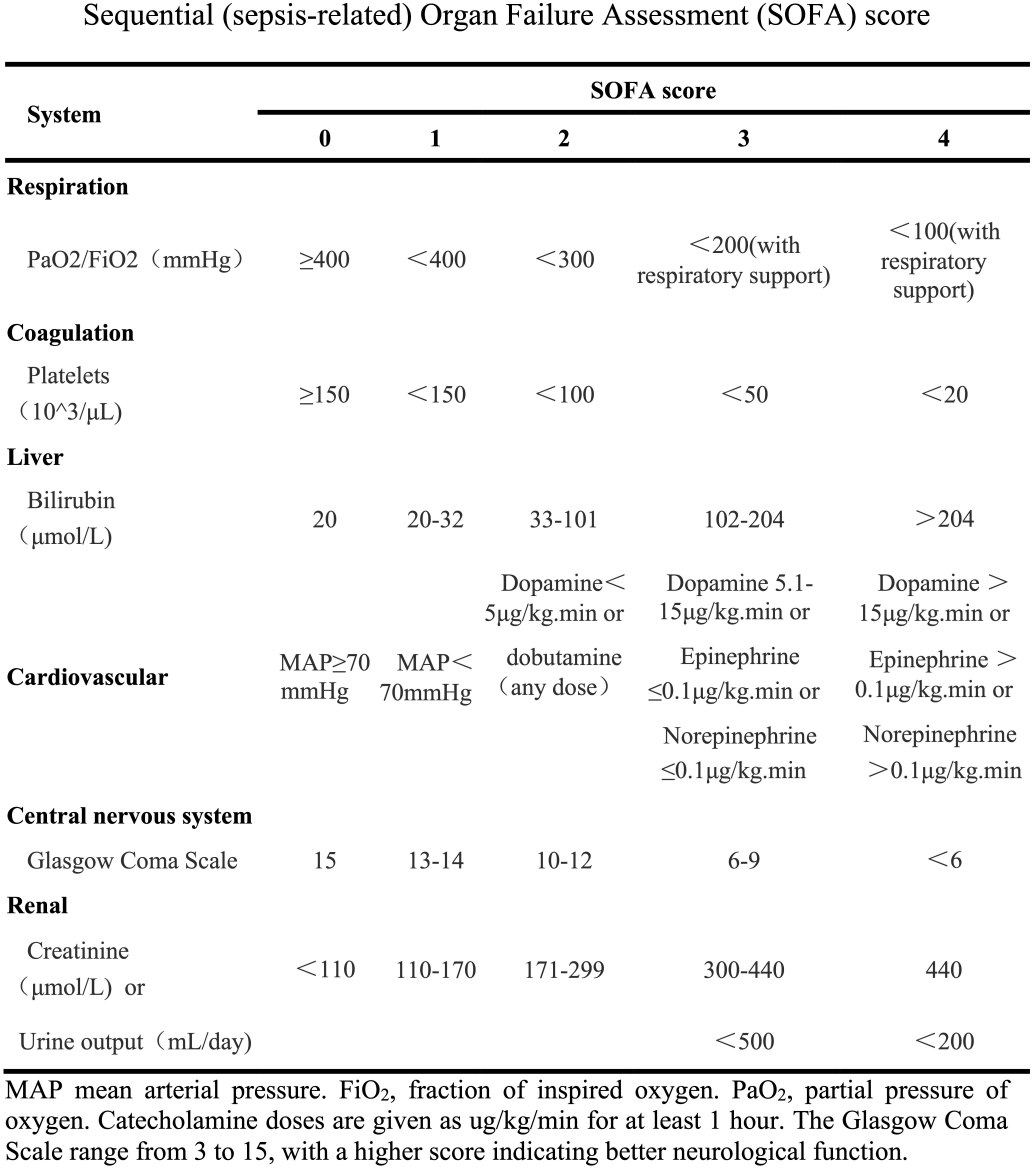
Sequential Organ Failure Assessment (SOFA) score for sepsis assessment

Clinical data of all cancer patients with sepsis were collected as follows (Table 1): (1) demographic characteristics: gender, age, and body mass index (BMI); (2) cancer types and treatment status; (3) chronic underlying diseases: hypertension, diabetes, coronary heart disease, chronic obstructive pulmonary disease, and cerebrovascular disease; (4) infection status: infection sites and etiology classification; (5) laboratory examination: leukocytes, neutrophils, lymphocytes, NLR, albumin, blood gas lactic acid, PCT, BNP, and cTnI on d1 and d3; and (6) others: fluid accumulation at 24 h and 72 h, SOFA score adapted from Vincent et al.^12^ (Figure 2) within 48 h of entering the ICU, sepsis‐related complications in ICU, sepsis‐associated intervention, the time of mechanical ventilation (MV), the time and the cost of staying in ICU.

**TABLE 1:** Comparison of clinical data between development cohort and validation cohort

Clinic data	Development cohort (*n* = 229)	Validation cohort (*n* = 88)	*p* value
Sex, male	172 (75.1%)	58 (65.9%)	0.13
Age(year)	63.5 ± 10.0	63.2 ± 11.0	0.83
BMI (kg/m^2^)	23.1 ± 4.9	23.3 ± 7.8	0.74
Cancer types			0.59
Lung	22 (9.6%)	13 (14.8%)	
Digestive system	160 (69.9%)	55 (62.5%)	
Retroperitoneum	18 (7.9%)	6 (6.8%)	
Uria	8 (3.5%)	3 (3.4%)	
Bone and soft tissue	1 (0.4%)	3 (3.4%)	
Gynecology	8 (3.5%)	3 (3.4%)	
Breast	3 (1.3%)	2 (2.3%)	
Lymphoma	2 (0.9%)	1 (1.1%)	
Melanoma	2 (0.9%)	1 (1.1%)	
Others	5 (2.2%)	1 (1.1%)	
Cancer treatment			
Surgery	156 (68.1%)	52 (70.5%)	0.69
Chemotherapy	86 (37.6%)	36 (40.9%)	0.58
Radiotherapy	35 (15.3%)	12 (13.6%)	0.71
Targeted therapy	44 (19.2%)	21 (23.9%)	0.39
Immunotherapy	25 (10.9%)	8 (9.1%)	0.63
Chronic diseases			
Hypertension	51 (22.3%)	15 (17.0%)	0.31
Diabetes	37 (16.2%)	19 (21.6%)	0.47
Coronary heart disease	33 (14.4%)	13 (14.8%)	0.94
COPD	34 (14.8%)	12 (13.6%)	0.78
Cerebrovascular disease	21 (9.2%)	7 (8.0%)	0.73
Infection category			0.12
Respiratory	77 (33.6%)	30 (34.1%)	
Gastrointestinal	11 (4.8%)	12 (13.6%)	
Abdominal cavity	107 (46.7%)	36 (40.9%)	
Thoracic cavity	24 (10.5%)	7 (8.0%)	
CLABSI	3 (1.3%)	0	
Genitourinary	5 (2.2%)	1 (1.1%)	
Others	2 (0.9%)	2 (2.3%)	
Organism			0.42
Gram negative	72 (31.4%)	34 (38.6%)	
Gram positive	33 (14.4%)	16 (18.2%)	
Fungi	22 (9.6%)	9 (10.2%)	
Two or more	48 (21.0%)	21 (23.9%)	
Laboratory examination(d1)			
Leukocyte(10^9^/L)	9.8 ± 6.0	9.0 ± 5.0	0.57
Neutrophil(10^9^/L)	8.2 (6.3–11.6)	7.8 (5.7–10.5)	0.49
Lymphocyte(10^9^/L)	0.6 (0.3–1.2)	0.7 (0.4–1.4)	0.46
NLR	11.6 (7.3–24.6)	10.6 (6.9–18.7)	0.39
Albumin(g/L)	32.2 ± 4.6	30.8 ± 4.5	0.28
Lactate(mmol/L)	2.9 ± 2.3	2.8 ± 2.3	0.92
PCT (ng/ml)	17.7 ± 46.9	14.5 ± 30.5	0.56
BNP (pg/ml)	658.6 ± 837.3	673.8 ± 835.2	0.88
cTnI (ng/ml)	0.03 (0.01–0.14)	0.02 (0.04–0.27)	0.10
Laboratory examination(d3)			
Leukocyte(10^9^/L)	12.2 ± 8.2	12.2 ± 7.5	0.97
Neutrophil(10^9^/L)	8.7 (5.6–13.2)	8.5 (6.3–12.8)	0.71
Lymphocyte(10^9^/L)	0.7 (0.4–0.9)	0.8 (0.5–1.1)	0.78
NLR	14.4 (10.1–18.9)	12.2 (7.9–18.4)	0.32
Albumin(g/L)	33.1 ± 3.1	32.2 ± 4.1	0.29
Lactate(mmol/L)	1.5 ± 1.3	1.9 ± 2.5	0.38
PCT (ng/ml)	8.6 ± 21.1	8.0 ± 20.4	0.75
BNP (pg/ml)	569.0 ± 738.2	553.8 ± 613.9	0.85
cTnI (ng/ml)	0.03 (0.01–0.12)	0.04 (0.02–0.13)	0.15
Fluid accumulation(ml/kg)			
At 24 h in ICU	47.4 ± 33.6	54.2 ± 39.6	0.91
At 72 h in ICU	64.2 ± 54.7	70.7 ± 46.8	0.37
SOFA score within 48 h in ICU	8 (6–11)	7 (6–10.5)	0.91
Sepsis‐associated complications			
Septic shock	104 (45.4%)	39 (44.3%)	0.86
AKI	47 (20.5%)	17 (19.3%)	0.81
ARF	113 (49.3%)	40 (45.4%)	0.68
SIMD^a^	44/144 (30.2%)	18/55 (37.3%)	0.92
Intervention for sepsis			
MV	99 (43.2%)	46 (52.3%)	0.15
CVVH	14 (6.1%)	6 (6.8%)	0.66
Emergency surgery due to sepsis	66 (28.8%)	29 (33.0%)	0.47
ICU MV time(day)	5.2 ± 2.6	4.9 ± 3.3	0.37
ICU stay time(day)	8.1 ± 8.1	8.3 ± 6.6	0.84
ICU cost ($)	8917 (3863.4–13612.0)	9102 (4312.2–13318.1)	0.76
The 28‐day mortality	40 (17.5%)	18 (20.5%)	0.18

*Note*: Data were expressed as mean ± standard deviation, number (percentage), or median (25th/75th percentile).

Abbreviations: AKI, acute kidney injury; ARF, acute respiratory failure; BMI, body mass index; BNP, brain natriuretic peptide; CLABSI, central line‐associated bloodstream infection; COPD, chronic obstructive pulmonary disease; cTnI, cardiac troponin I; CVVH, Continuous Veno‐Venous Hemodialysis; d1, the first day after entering ICU; d3, the third day after entering ICU; ICU, intensive care unit; MV, mechanical ventilation; NLR, Neutrophil‐to‐Lymphocyte Ratio; PCT, Procalcitonin; SIMD, sepsis‐induced myocardial dysfunction; SOFA, Sequential Organ failure assessment.

^**a**^ One hundred and forty four patients in the development cohort and fifty five patients in the validation cohort underwent bedside echocardiography.

### 2.3 Statistics

All data were expressed as the mean ± standard deviation, number (rate), and median (interquartile range). Normally or skewed distributed continuous variables were analyzed with the unpaired *t* test or Mann–Whitney *U* test, respectively. Categorical variables were compared by a χ^2^ test. The independent risk factors were screened out with multivariable logistic regression analysis. A nomogram was established based on these independent risk factors in the development cohort. Consistency (C) statistics were used to evaluate the discriminative ability of the nomogram. A calibration chart reflected the consistency between the predicted probability and the actual probability. The receiver operating characteristic (ROC) curve was used to further evaluate the effectiveness of the nomogram in predicting the 28‐day mortality due to sepsis in ICU, and the best cutoff value was determined according to the Youden index. SPSS version 26.0 (IBM) and R version 4.1 were used for statistical analysis. A two‐sided *p* < 0.05 was considered statistically significant.

## 3 RESULTS

**FIGURE 3:**
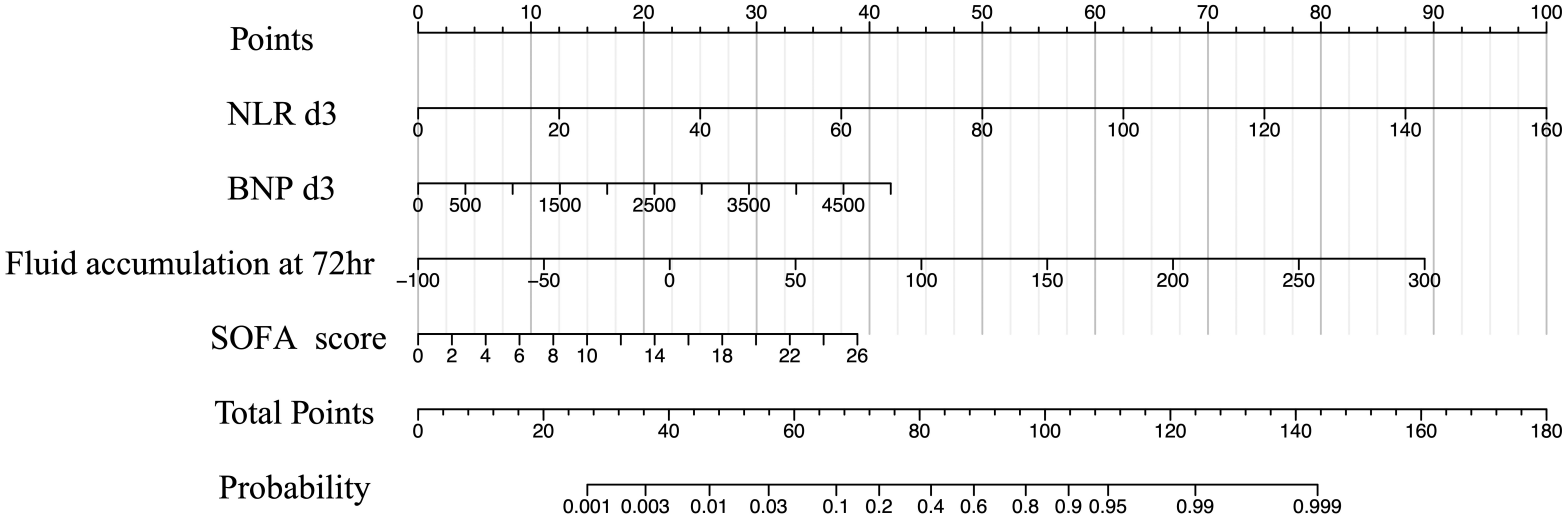
A nomogram established from the development cohort for predicting the 28‐day mortality of cancer patients with sepsis

**FIGURE 4:**
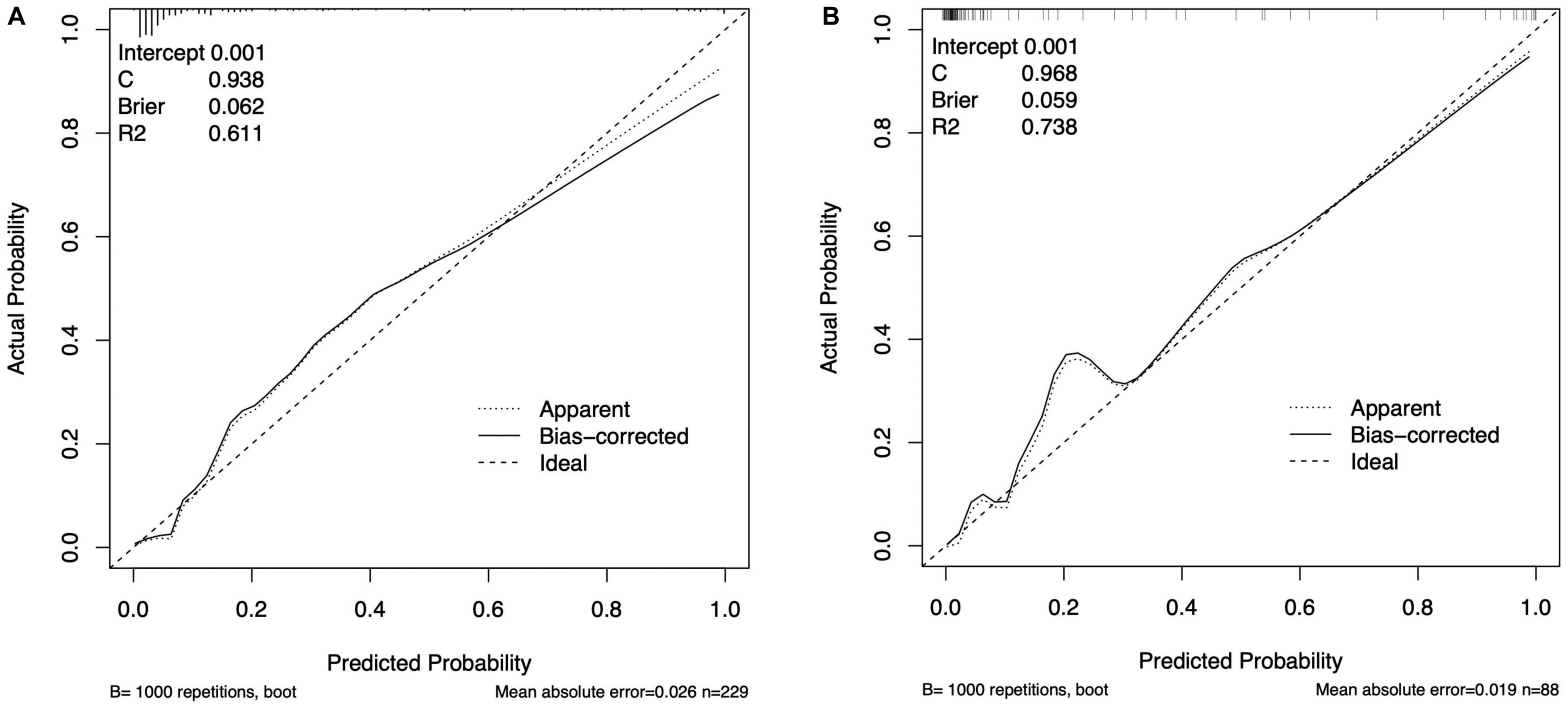
Calibration plots in the development cohort (A) and the validation cohort (B)

**FIGURE 5:**
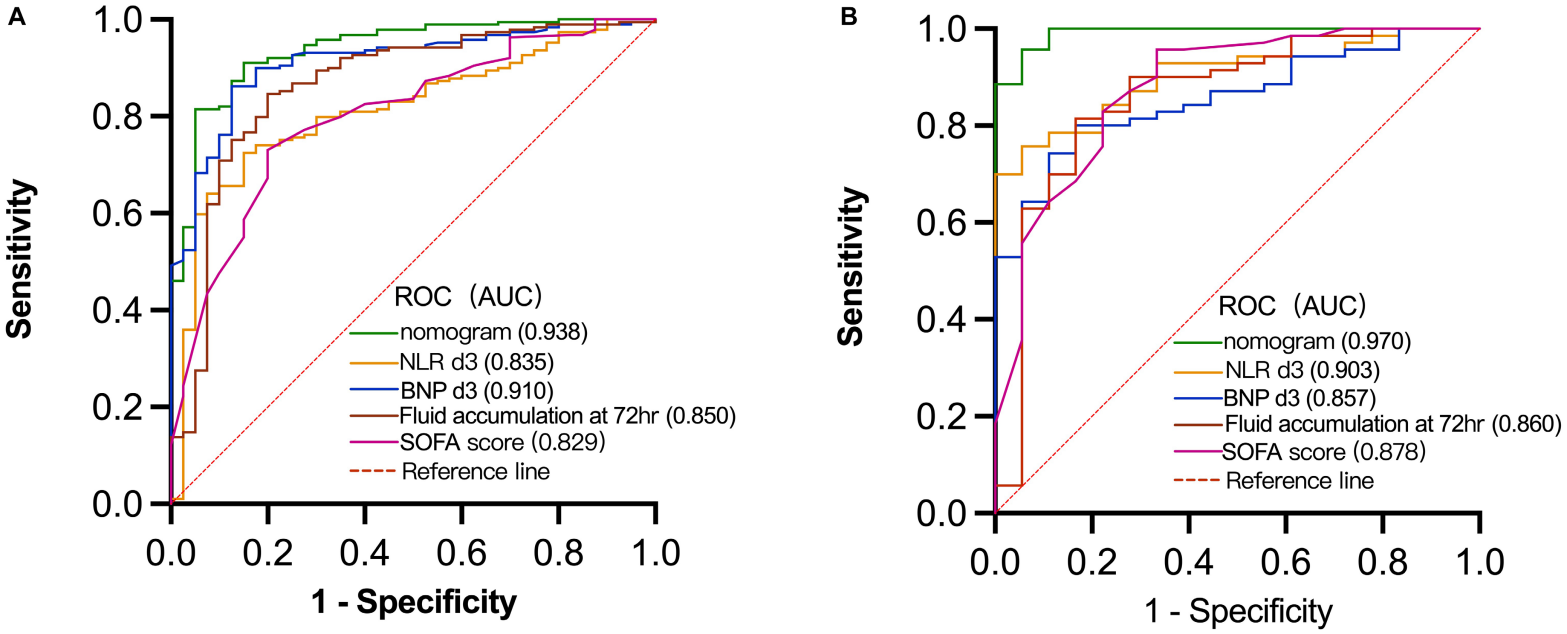
AUCs of the nomogram, NLR d3, BNP d3, fluid accumulation at 72 h and SOFA score, in the development cohort (A) and validation cohort (B) with ROC analysis

1. The clinical data of patients between the development cohort (*n* = 229) and the validation cohort (*n* = 88) were well comparable (*p* > 0.05). The 28‐day mortality rates were 17.5% (40/229) and 20.5% (18/88), respectively (Table 1).
2. The univariate analysis results of patients in the survivors (*n* = 189) and the nonsurvivors (*n* = 40) in the development cohort are shown in Table 2. Multivariate analysis showed that NLR d3, BNP d3, fluid accumulation at 72 h, and SOFA score were independent risk factors for the 28‐day mortality due to sepsis in the development cohort (Table 2).
3. The equation expression of a prediction model for mortality due to sepsis based on logistic multivariate regression analysis: logit(**P**) = −6.768 + 0.048*NLR d3 + 0.003*BNP d3 + 0.023*fluid accumulation at 72 h + 0.134*SOFA (**P** means the probability of the 28‐day mortality due to sepsis). The equation can be deformed to **P** = 1/(1 + e ‐ logit(**P**)). The Hosmer and Lemeshow test indicates that the model fits well. The optimal cutoff values for NLR d3, BNP d3, fluid accumulation at 72 h, and SOFA score were 16.4, 604.5 pg/ml, 88.7 ml/kg, and 8.75, respectively. The above cutoff values were substituted into the deformed equation, and **P** was calculated (**P** = 0.14).
4. A nomogram was established based on the above equation (Figure 3). The calibration plot of the nomogram revealed an adequate fit in the development cohort (Figure 4A) and the validation cohort (Figure 4B) according to the predicted probability or the actual probability, which also had good statistical consistency in predicting the 28‐day mortality due to sepsis (the *C* value was 0.938 and 0.968 in the two cohorts, respectively). The optimal cutoff score of the nomogram for predicting 28‐day mortality was 83.8 points in the development cohort, which was consistent with **P** in the logistic regression equation. With a threshold of 83.8 points, patients in the development cohort and validation cohort were predicted to be survivors or non‐survivors. Compared with the observed outcome, the nomogram had an accuracy rate of 90.8%, a sensitivity of 72.5%, a specificity of 94.7%, a positive predictive value of 72.3%, and a negative predictive value of 94.2% for predicting mortality in the development cohort (Table 3). It also had an accuracy rate of 92.0%, a sensitivity of 77.7%, a specificity of 95.7%, a positive predictive value of 82.4%, and a negative predictive value of 94.4% for predicting mortality in the validation cohort (Table 4).
5. The discriminatory ability of the nomogram for predicting 28‐day mortality was evaluated by AUCs with ROC curve analysis. The nomogram in the development cohort had an AUC of 0.938 (95% CI: 0.915–0.984), which was significantly larger than that of the NLR d3 (0.835, 95% CI: 0.778–0.896), BNP d3 (0.910, 95% CI: 0.867–0.962), and fluid accumulation in 72 h (0.850, 95% CI: 0.772–0.928), and SOFA score (0.829, 95% CI: 0.736–0.891) (all *p* < 0.001) (Figure 5A), showing good statistical value for prediction of the 28‐day mortality. The discriminatory ability was also good in the validation cohort, with an AUC of 0.970 (95% CI: 0.933–1.000), which was significantly larger than that of the NLR d3 (0.903, 95% CI: 0.808–0.956), BNP d3 (0.857, 95% CI: 0.812–0.902), and fluid accumulation at 72 h (0.860, 95% CI: 0.791–0.913) and SOFA score (0.878, 95% CI: 0.836–0.970) (all *p* < 0.001) (Figure 5B).

**TABLE 2:** Univariable and multivariable logistic regression between survivors and nonsurvivors in the development cohort

Variables	Univariable logistic regression		Multivariable logistic regression	
	Odds ratio (95% CI)	*p value*	Odds ratio (95% CI)	*p value*
Sex	0.768 (0.314–1.725)	0.57	—	—
Age	1.026 (0.990–1.068)	0.21	—	—
BMI	1.128 (1.036–1.224)	0.02*	0.665 (0.542–1.121)	0.47
Leucocyte d1	0.968 (0.844–1.147)	0.76	—	—
Neutrophil d1	0.976 (0.824–1.157)	0.74	—	—
Lymphocyte d1	2.128 (0.147–26.781)	0.59	—	—
NLR d1	0.944 (0.855–1.043)	0.26	—	—
Albumin d1	0.987 (0.815–1.197)	0.87	—	—
Lactate d1	1.001 (1.000–1.016)	0.03*	0.622 (0.481–1.202)	0.86
PCT d1	1.011 (0.986–1.023)	0.09	—	—
BNP d1	1.000 (1.000–1.001)	0.04*	0.781 (0.761–1.304)	0.34
cTnI d1	1.021 (0.962–1.084)	0.39	—	—
Leukocyte d3	0.986 (0.916–1.034)	0.36	—	—
Neutrophil d3	1.041 (0.921–1.112)	0.21	—	—
Lymphocyte d3	0.528 (0.191–1.294)	0.17	—	—
NLR d3	1.103 (1.052–1.154)	<0.001*	1.093 (1.019–1.172)	0.013*
Albumin d3	0.568 (0.698–1.012)	0.36	—	—
Lactate d3	2.031 (0.919–4.489)	0.08	—	—
PCT d3	1.066 (0.978–1.161)	0.15	—	—
BNP d3	1.003 (1.002–1.004)	<0.001*	1.004 (1.002–1.006)	<0.001*
cTnI d3	1.132 (1.031–1.243)	0.04*	0.813 (0.738–1.301)	0.76
Fluid accumulation at 24 h in ICU	1.011 (1.001–1.021)	0.12	0.719 (0.692–1.102)	0.65
Fluid accumulation at 72 h in ICU	1.020 (1.011–1.022)	<0.001*	1.017 (1.005–1.029)	0.003*
SOFA score	1.313 (1.201–1.405)	<0.001*	1.262 (1.059–1.511)	0.005*

*Note*: * represented *p* < 0.05.

Abbreviations: BMI, body mass index; BNP, brain natriuretic peptide; cTnI, cardiac troponin I; d1, the first day after entering ICU; d3, the third day after entering ICU; NLR, Neutrophil‐to‐Lymphocyte Ratio; PCT, Procalcitonin; SOFA, Sequential Organ failure assessment.

**TABLE 3:** Prediction of nomogram in development cohort

Predicted outcome (nomogram)	Actual outcome		Total
	Nonsurvivors	Survivors	
Nonsurvivors	29	10	39
Survivors	11	179	190
Total	40	189	229

**TABLE 4:** Prediction of nomogram in Validation cohort

Predicted outcome (nomogram)	Actual outcome		Total
	Nonsurvivors	Survivors	
Nonsurvivors	14	3	17
Survivors	4	67	71
Total	18	70	88

## 4 DISCUSSION

Sepsis is widespread in critically ill patients in the ICU.^13^ Relative to patients without cancer, the combined risk of sepsis in cancer patients is the former 10 times.^14^ Cancer patients with sepsis are part of the main reasons for entering the ICU.^15^ In a series of recent studies, when cancer patients in the ICU had sepsis, the mortality rate varied from 20% to 60%.^16^, ^17^ For the special population of cancer patients with sepsis, it is very important to find some simple and sensitive clinical variables to predict the risk of mortality from sepsis. Early active intervention is necessary.^18^

Because of the complex pathogenesis of sepsis, multiple variables have continuously emerged in the field of prognostic judgment of sepsis. An elevated C‐reactive protein/albumin ratio (CRP/ALB) 72 h after admission indicates an increased risk of mortality in patients with sepsis.^19^ The level of plasma interleukin‐33 (IL‐33) is positively correlated with the severity of sepsis.^20^ Elevated serum heparin‐binding protein is a risk factor for early mortality in patients with sepsis.^21^ However, some variables are difficult to obtain in clinical application, and some have varying degrees of defects in accuracy, sensitivity, or specificity. In addition, clinicians have different experiences and preferences in their subjective judgments on prognostic variables of sepsis. The above factors ultimately lead to inaccurate judgments on the prognosis of sepsis.^22^ The search for more convenient and accurate prognostic variables of sepsis has never stopped.

Multivariate logistic regression analysis showed that NLR d3, BNP d3, fluid accumulation at 72 h, and SOFA score were independent risk factors in this study. The NLR has a good prognostic value in sepsis. Liu et al. found that NLR after entering the ICU was closely related to the 28‐day mortality of sepsis and the severity of the disease.^23^ The NLR d3 of nonsurvivors was significantly higher than that of survivors in the development cohort of this study (*p* < 0.05). Rehman et al. found that if patients with sepsis worsened after entering the ICU, the NLR value increased more dramatically. They also found that NLR on the third day after entering the ICU was an independent risk factor for mortality in patients with sepsis,^24^ which is consistent with our study. However, Salciccioli et al. believe that there was no obvious correlation between NLR and mortality of sepsis.^25^ This study concluded that BNP d3 was one of the independent risk factors for mortality in patients with sepsis. Multiple studies have also shown that BNP is closely related to 30‐day and 90‐day all‐cause mortality in patients with sepsis.^26^ BNP is kept in the ventricular septum. The variable myocardial contractility produced by sepsis causes BNP to be released into the blood in large amounts, exerting its physiological effects such as blood vessel expansion and diuresis. Its metabolism is influenced by age and renal function.^27^ Although the conclusion of the development cohort of this study suggests that BNP d3 in the ICU is 604.5 pg/ml as the cutoff value, the AUC for predicting mortality of sepsis is 0.910, with a sensitivity of 89% and a specificity of 86%.The overall predictive performance of BNP d3 is excellent. However, there are many confounding factors of BNP, and a single variable is not accurate in judging the prognosis of sepsis. Fluid accumulation at 72 h in the ICU constituted one of the independent risk factors. Fluid resuscitation in the early stage of sepsis is imperative. However, persistent fluid balance in the later stage of sepsis is associated with a poor prognosis. The initial Sepsis Occurrence in Acutely ill Patients (SOAP) study shows that the amount of fluid accumulation within 72 hours is a strong predictor of mortality in patients with sepsis in the ICU.^28^ The Vasopressin and Septic Shock Trial (VASST) study also clarified that there was a significant correlation between fluid accumulation within 4 days after entering the ICU and the mortality of patients with sepsis.^29^ Some patients in our study underwent emergency surgery and required rapid rehydration during surgery. Therefore, the amount of fluid accumulation within 3 days of entering the ICU was not entirely due to septic fluid resuscitation, and there was a certain bias. The SOFA score is an important part of the definition of sepsis 3.0, which has satisfactory reliability in assessing the severity of illness in critically ill patients.^30^ However, many studies have demonstrated that the sensitivity and specificity of SOFA scores are relatively low.^31^, ^32^

The above single variables have their limitations. Therefore, fitting multiple simple and sensitive variables to generate joint predictors to judge the prognosis of sepsis has become a current research hotspot.^33^ Fitting multiple single variables to construct a new joint prediction method with logistic regression was first reported by the statistician Pepe.^34^ The nomogram established according to the logistic regression equation statistically quantifies and visualizes the contribution of every single variable. It can eliminate the possible confounding factors between single variables and make the judgment of prognosis more objective and comprehensive.^35^ The nomogram based on the four independent risk factors with logistic regression in the development cohort has good predictive effectiveness for the 28‐day prognosis due to sepsis in this study. After multiple verifications, its predictive performance was better than that of anyone independent risk factor.

This study has certain limitations. First, this is a retrospective study, and the overall sample size for modeling was relatively small. Second, most of the independent risk factors obtained are data on the 3rd day after the patient enters the ICU, with a time lag. The nomogram established on this basis is not appropriate for patients who died of sepsis within 3 days after entering the ICU. Third, the sample size of sepsis patients in the validation cohort was small. Building on the above situation, we need to find more variables and expand the sample size to build an earlier and more accurate nomogram for the prognosis of cancer patients with sepsis.

## 5 CONCLUSION

This study screened out independent risk factors for 28‐day mortality in cancer patients with sepsis. The nomogram established based on logistic regression fitting all independent risk factors has good predictive power for the 28‐day prognosis due to sepsis after preliminary verification. The nomogram is simple and easy to use. More sample sizes can be tried in clinical work to verify the prognostic ability of the nomogram.

## CONFLICT OF INTEREST

All authors declare that they have no conflicts of interest and have never published the manuscript.

## AUTHOR CONTRIBUTIONS

(I) Conception and design: Y Yang; (II) Administrative support: Hz Wang, Cy Hao; (III) Provision of study materials or patients: Y Yang, Jun D; (IV) Collection and assembly of data: Y Yang, Rx Chen; (V) Data analysis and interpretation: Y Li, Xy Tian; (VI) Manuscript writing: All authors; (VII) Final approval of manuscript: All authors.

## ETHICS APPROVAL

The study complies with the Declaration of Helsinki and was approved by the Ethics Committee of Peking University Cancer Hospital. All patients admitted to ICU signed an informed consent form for the treatment of sepsis and scientific purposes.

## Data Availability

The datasets used and analyzed during the current study are available from the corresponding author on reasonable request.
